# Solution structure of the RNA recognition domain of METTL3-METTL14 N^6^-methyladenosine methyltransferase

**DOI:** 10.1007/s13238-018-0518-7

**Published:** 2018-03-14

**Authors:** Jinbo Huang, Xu Dong, Zhou Gong, Ling-Yun Qin, Shuai Yang, Yue-Ling Zhu, Xiang Wang, Delin Zhang, Tingting Zou, Ping Yin, Chun Tang

**Affiliations:** 1 National Key Laboratory of Crop Genetic Improvement and National Center of Plant Gene Research, Huazhong Agricultural University, Wuhan, 430070 China; 2 CAS Key Laboratory of Magnetic Resonance in Biological Systems, State Key Laboratory of Magnetic Resonance and Atomic Molecular Physics, and National Center for Magnetic Resonance at Wuhan, Wuhan Institute of Physics and Mathematics of the Chinese Academy of Sciences, Wuhan, 430071 China

**Keywords:** RNA modification, N^6^-methyladenosine, METTL3, target recognition domain, zinc finger, paramagnetic relaxation enhancement

## Abstract

N^6^-methyladenosine (m^6^A), a ubiquitous RNA modification, is installed by METTL3-METTL14 complex. The structure of the heterodimeric complex between the methyltransferase domains (MTDs) of METTL3 and METTL14 has been previously determined. However, the MTDs alone possess no enzymatic activity. Here we present the solution structure for the zinc finger domain (ZFD) of METTL3, the inclusion of which fulfills the methyltransferase activity of METTL3-METTL14. We show that the ZFD specifically binds to an RNA containing 5′-GGACU-3′ consensus sequence, but does not to one without. The ZFD thus serves as the target recognition domain, a structural feature previously shown for DNA methyltransferases, and cooperates with the MTDs of METTL3-METTL14 for catalysis. However, the interaction between the ZFD and the specific RNA is extremely weak, with the binding affinity at several hundred micromolar under physiological conditions. The ZFD contains two CCCH-type zinc fingers connected by an anti-parallel β-sheet. Mutational analysis and NMR titrations have mapped the functional interface to a contiguous surface. As a division of labor, the RNA-binding interface comprises basic residues from zinc finger 1 and hydrophobic residues from β-sheet and zinc finger 2. Further we show that the linker between the ZFD and MTD of METTL3 is flexible but partially folded, which may permit the cooperation between the two domains during catalysis. Together, the structural characterization of METTL3 ZFD paves the way to elucidate the atomic details of the entire process of RNA m^6^A modification.

**Electronic supplementary material:**

The online version of this article (10.1007/s13238-018-0518-7) contains supplementary material, which is available to authorized users.

## INTRODUCTION

N^6^-methyladenosine (m^6^A) is a ubiquitous RNA epigenetic mark, and has been identified in a large number of coding and non-coding regions of RNA molecules (Fu et al. 2014; Liu and Pan 2016; Roundtree et al. 2017). The m^6^A modification is involved in a multitude of physiological and pathophysiological processes, including sex determination (Lence et al. 2016), cell differentiation (Yoon et al. 2017), DNA damage response (Xiang et al. 2017), viral infection (Gokhale et al. 2016) and innate immunity (Zheng et al. 2017). The m^6^A modification occurs to RNAs containing 5′-RRACH-3′ sequence, in which the nucleotide A_3_ becomes N^6^-methylated (Schibler et al. 1977; Dominissini et al. 2012; Song and Yi 2017). The m^6^A modification is installed by a multi-subunit complex that comprises the heterodimer of METTL3 and METTL14 (Liu et al. 2014; Wang et al. 2014) and accessory proteins including WTAP (Zhong et al. 2008; Ping et al. 2014), KIAA1429 (Schwartz et al. 2014) and RBM15 (Patil et al. 2016).

No structural information is available for the full-length METTL3, METTL14 and their complexes. The structure of the methyltransferase domain (MTD) of METTL3-METTL14 heterodimer has been determined using X-ray crystallography (Sledz and Jinek 2016; Wang et al. 2016a, b). The MTD construct of METTL3 encompasses residues 358–580, while the MTD of METTL14 comprises residues 110–404. The structure shows that the MTD of METTL3 binds to S-adenosyl methionine (SAM) and therefore functions as the catalytic core. The structure of METTL14 MTD is similar to that of METTL3 MTD, but it lacks the SAM binding site and may only stabilize the structure of the heterodimer. Nevertheless, the MTD heterodimer of METTL3-METTL14 used in previous structural studies has no methyltransferase activity (Sledz and Jinek 2016; Wang et al. 2016a; Wang et al. 2017). This means that domains besides the MTD should be involved for catalysis.

Two CCCH-type zinc fingers have been identified in METTL3 to the N-terminus of the MTD (Iyer et al. 2016), but not in METTL14. CCCH-type zinc fingers are known involved in RNA binding and RNA metabolism (Amann et al. 2003; Murn et al. 2016; Fu and Blackshear 2017). Here we report the solution structure of METTL3 zinc finger domain (ZFD) that comprises two tandem zinc fingers. We show that the ZFD is primarily responsible for specific binding to m^6^A RNA substrate, and thus serves as the target recognition domain and fulfills the methyltransferase activity of METTL3-METTL14 complex.

## RESULTS

### ZFD is responsible for specific RNA recognition

**Figure 1:**
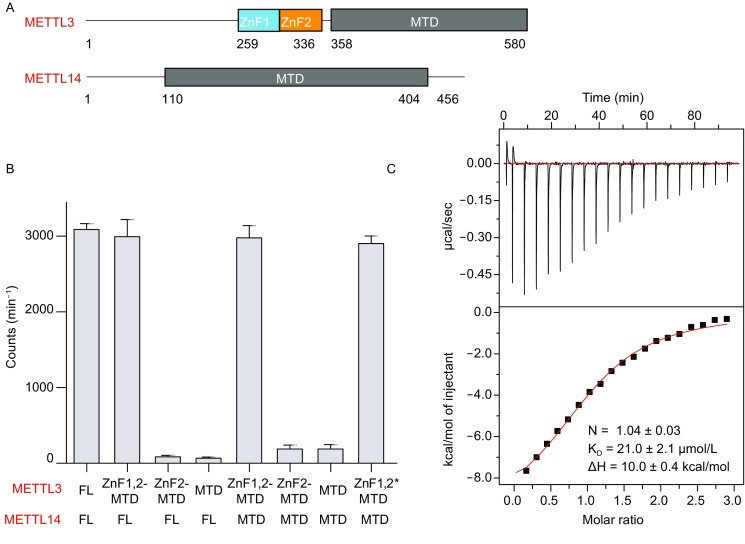
**The ZFD is required for METTL3-METTL14 methyltransferase activity**. (A) Domain architecture of METTL3 and METTL14. The structure for the MTD heterodimer has been previously determined. (B) Methyltransferase activities for various constructs of METTL3-METTL14. The ZFD of METTL3 is either recombinantly expressed (denoted with a -) or natively ligated to the MTD (denoted with a *). The average counts per minute (±SD with *n* = 3) are reported. (C) Isothermal calorimetry measurement with 1.5 mmol/L specific RNA containing the 5′-GGACU-3′ consensus sequence titrated to 70 µmol/L ZFD. Averaged binding and thermodynamics parameters are reported from triplicated experiments

The ZFD domain in the METTL3 contains two CCCH-type zinc fingers, namely ZnF1 and ZnF2. ZnF1 and ZnF2 roughly encompass residues 259–298 and 299–336 (Fig. 1A), and their sequences are largely conserved across species (Fig. S1). We assessed the methyltransferase activity for the heterodimer between METTL3 (including both the ZFD and MTD, and spanning residues 259–580) and METTL14. Be it the full-length METTL14 or only the MTD of METTL14, the resulting complex displays methyltransferase activity as high as the heterodimer of the full-length METTL3-METTL14 (Fig. 1B). Furthermore, both zinc fingers in the ZFD are required for METTL3-METTL14 methyltransferase activity, as the heterodimer loses its activity if the ZnF1 is not included in the construct. Thus the ZFD of METTL3 complements the MTD in installing the m^6^A onto target RNA molecules.

Zinc fingers have been shown involved in RNA binding (Park et al. 2017). To assess the interaction between METTL3 ZFD and RNA, we prepared the ZFD construct (residues 259–357) and performed isothermal calorimetry (ITC) titrations. At 37°C, the interaction between ZFD and an RNA molecule containing the consensus recognition sequence 5′-GGACU-3′ flanked by adenosines was too weak to be accurately assessed by ITC (Fig. S2A). Fortunately, the *K*_d_ value could be determined at a lower temperature; at 10°C, the heat exhausted could be fitted to a one-site binding model with a *K*_d_ value of 21.0 ± 2.1 µmol/L (Fig. 1C). The binding affinity is about the same regardless of the pH and buffer condition (Fig. S3A). In comparison, a nonspecific RNA molecule containing only adenosines does not bind to the ZFD, as assessed by the ITC also at 10°C (Fig. S2B). On the other hand, the interaction between the heterodimer of the METTL3-METTL14 MTD and the 5′-GGACU-3′ containing RNA molecule was also undetectable (Fig. S2C), while the enzymatically active heterodimer of METTL3 ZFD-MTD and METTL14 MTD binds to the specific RNA with about the same affinity as the METTL3 ZFD alone (Fig. S2D). Together, the METTL3 ZFD is responsible for the specific interaction between METTL3-METTL14 and its RNA substrate, thus enabling the methyltransferase activity.

### Solution structure of METTL3 ZFD

**Figure 2:**
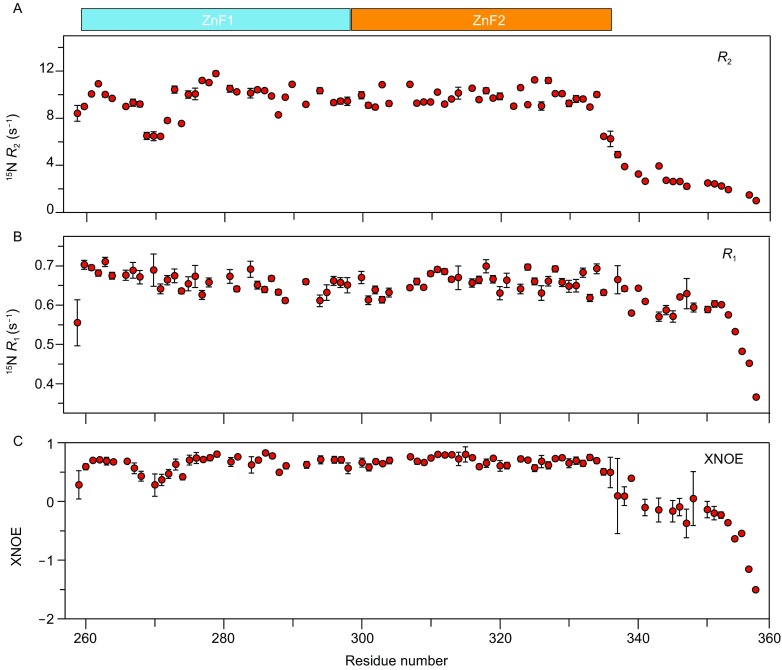
**Relaxation analysis for backbone amide nitrogen atoms of METTL3 ZFD**. (A) Transverse relaxation rates *R*_2_, (B) longitudinal relaxation rates *R*_1_, (C) ^15^N heteronuclear NOE (XNOE). Though the *R*_2_ and XNOE data indicate that residues 259–336 are mostly rigid, the *R*_1_ and XNOE data also indicate that residues 337–353 in the C-terminal tail also retain some order and tumble at sub-ns to ns timescale. The errors bars indicate the SD calculated from measurement uncertainties

To appreciate the functional significance of METTL3 ZFD, we characterized its solution structure using NMR. Using a set of through-bond NMR experiments, we were able to assign over 95% of backbone and side chain resonances for METTL3 ZFD. We measured the relaxation parameters for protein backbone amide ^15^N atoms (Kay et al. 1989; Kleckner and Foster 2011). Transverse relaxation rates *R*_2_ for amide ^15^N atoms indicates that ZFD residues 337–357 are dynamic at ps-ns timescale (Fig. 2A). In comparison, the longitudinal relaxation rates *R*_1_ do not significantly decrease until after residue 353 (Fig. 2B). The heteronuclear NOE (XNOE) values for ZFD residues 337–357 are <0.5 (Fig. 2C), thus confirming that the C-terminal residues are very dynamic. However, the XNOE values for the C-terminal tail do not fall below −0.5 until after residue 353. Together the *R*_1_ and XNOE values indicate that residues 337–353 retain some order and mainly fluctuate at sub-ns to ns timescale.

As a flexible linker connects the ZFD and MTD of METTL3, we prepared a segmentally ^15^N-labeled ZFD natively ligated to the unlabeled MTD heterodimer of METTL3-METTL14. With the ZFD and MTD fused together, the heterodimer of METTL3 and METTL14 displays almost identical methyltransferase activity as the wild-type recombinant full-length heterodimer (Fig. 1B). Significantly, the HSQC spectrum for the segmentally labeled ZFD can be readily visualized without much suffering to line broadening upon the increase of molecular weight, and can be nicely overlaid onto the spectrum of the isolated ZFD, with the exception of a few residues near the ligation site (Fig. S4A). As such, the METTL3 ZFD adopts the same structure when it is part of the catalytically active heterodimer or by itself, and therefore a divide-and-conquer approach to characterize the structure of the ZFD is justified.

**Figure 3:**
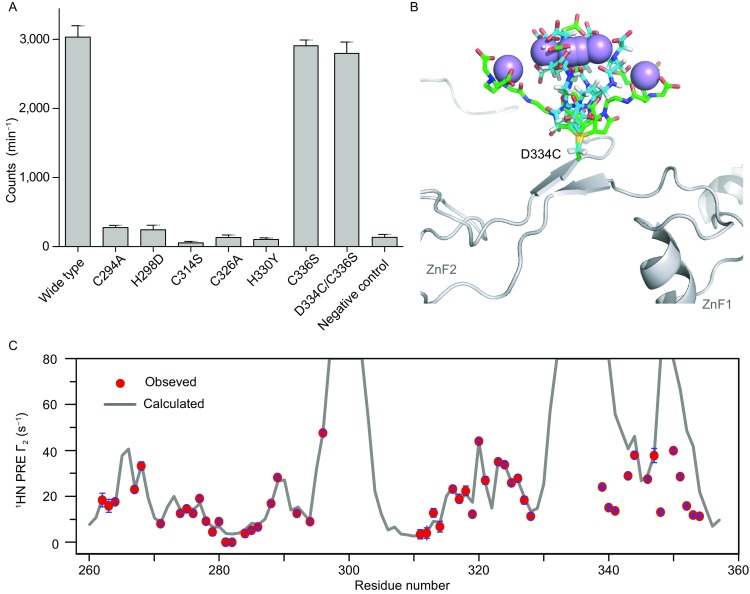
**Structure analysis of METTL3 ZFD**. (A) Point mutations introduced to the Zn^2+^-coordinating residues render the METTL3-METTL14 methyltransferase inactive. In comparison, mutation to the non-essential cysteine C336 has no effect. C314S and H330Y mutations have been identified in cancer patients. The average counts per minute (±SD with *n* = 3) are reported. (B) Paramagnetic probe maleimide-EDTA-Mn^2+^ conjugated at D334C site of the ZFD. The probe is represented with 6 conformers shown as green and cyan sticks, 3 for each configuration upon Michael addition, respectively. The conformers are optimized with torsion angle dynamics during protein structure refinement. The metal ions are shown as spheres. (C) The PRE measurement provides long-range structural constraints. The observed values are shown as red circles, with the error bars denoting the SD from measurement uncertainties. The back-calculated values from the final structure closest-to-mean are shown as black line

Residues C276, C284, C294 and H298 in ZnF1 and residues C314, C320, C326 and H330 in ZnF2 are highly conserved (Fig. S1) and should be involved in Zn^2+^-coordination in both CCCH-type zinc fingers (Iyer et al. 2016; Wang et al. 2016). Other cysteines and histidines in the ZFD, including His306, His322, Cys336 and His347 cannot satisfy the geometrical requirement of CCCH-type zinc-finger (Fu and Blackshear 2017). Furthermore, mutations to the Zn^2+^-coordinating residues, including C294A, H298D, C314S, C326A and H330Y, nearly abolish the methyltransferase activity, whereas a C336S mutation has no effect (Fig. 3A). Note that C314S and H330Y mutations have been identified in cancer patients (Tomczak et al. 2015). To assess how H298 and H330 coordinate Zn^2+^ atoms, we performed long-range ^1^H-^15^N HSQC experiment (Pelton et al. 1993). The spectrum indicates that both H298 and H330 should exist in δ1-H neutral tautomeric state (Fig. S4B), and use ε2 nitrogens of the histidines for Zn^2+^-coordination.

To determine the solution structure of METTL3 ZFD, we collected NOE, backbone dihedral angle, small-angle X-ray scattering (SAXS) and paramagnetic relaxation enhancement (PRE) restraints. The PRE measurement provides the long-range distance relationship between protein nuclei and paramagnetic center (Liu et al. 2015), and was obtained upon site-specific conjugation of a maleimide-EDTA-Mn^2+^ probe (Liu et al. 2012) to D334C/C336S double mutant of the ZFD. Probably due to its bulkiness and charge, the paramagnetic probe was found only conjugated at D334C as evidenced from the overall mass. Importantly, the METTL3/METTL14 heterodimer harboring D334C/C336S mutations has no loss of activity (Fig. 3A), and the conjugation of the paramagnetic probe causes no further chemical shift perturbations.

**Figure 4:**
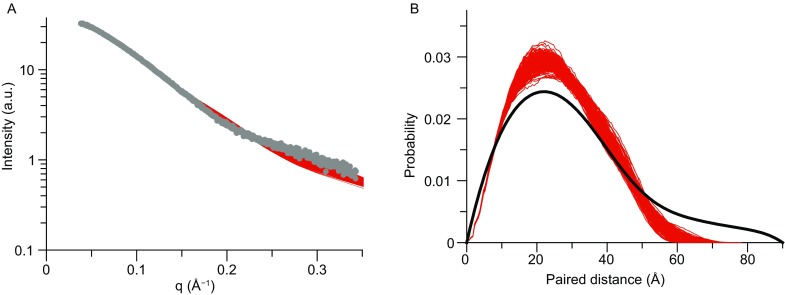
**Analysis of METTL3 ZFD structure with small-angle X-ray scattering**. (A) Comparison between the experimental (shown as gray dots for the truncated version of ZFD for residues 258–338) and calculated (shown as red lines) scattering profiles. The scattering curves are calculated for all 25 conformers in the bundle. (B) Comparison between the experimental (shown as black line) and calculated (shown as red lines) paired distance distribution P(r) functions. The P(r) profile suggests that the C-terminal tail of the ZFD likely interconverts between open and closed conformations

We refined the structure of METTL3 ZFD against the multiple types of restraints as well as knowledge-based restraints to enforce Zn^2+^-coordination geometry (Peters et al. 2010). The resulting structure can account for all experimental data, while the different types of restraints cross-validate each other. With the ensemble-representation for the paramagnetic probe conjugated at D334C site (Fig. 3B), the back-calculated PRE values for backbone amide protons are highly correlated to the observed values, with the average Q-factor of 0.09 (Figs. 3C and S5). In addition, the back-calculated SAXS curve for the rigid portion of the ZFD agrees well the observed with the observed data with the average *χ*^2^ value of 5.9 (Fig. 4A).

**Figure 5:**
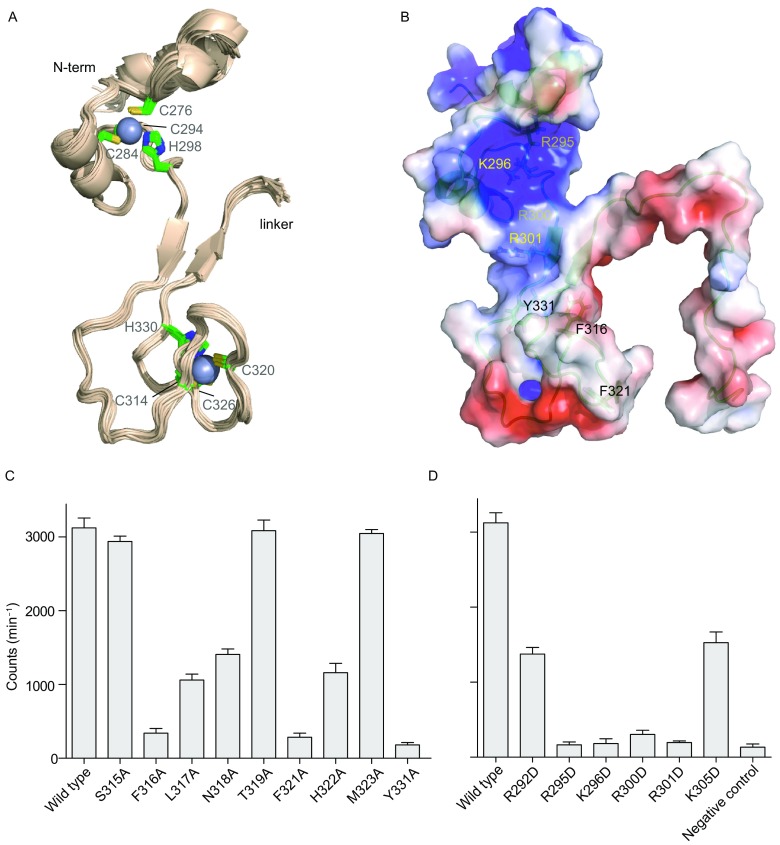
**Solution structure of METTL3 ZFD**. (A) Superposition of 25 lowest-energy structures of the ZFD. The structures were superimposed by backbone heavy atoms for residues 276–336, and the last 20 flexible residues are not shown. Zn^2+^ ions are shown as spheres, and the coordinating residues are shown as sticks. (B) Electrostatic potential colored surface, on a scale from red (−3 kT/e) to blue (+4 kT/e), with the functionally important basic residues (yellow labels) and hydrophobic residues (black labels) denoted. (C) Methyltransferase activities for the METTL3-METTL14 heterodimers harboring point mutations to the hydrophobic residues. (D) Methyltransferase activities for the METTL3-METTL14 heterodimers harboring point mutations to the basic residues. Negative control (NC) was assessed for an RNA containing 5′-GGGCU-3′ instead of 5′-GGACU-3′ consensus sequence. The average counts per minute (±SD with *n* = 3) are reported

The structures calculated for the ZFD are well converged (Fig. 5A). The root-mean-square (RMS) deviation of all residues is 0.90 ± 0.20 Å for backbone heavy atoms, and 1.22 ± 0.16 Å for all heavy atoms (Table 1). The two zinc fingers in the ZFD are connected by a two-stranded anti-parallel β-sheet that comprises residues 300–302 and 332–334. The ZnF1 is more positively charged than the ZnF2, while the ZnF2 has a patch of hydrophobic surface that mostly comprises residues 315–323 (Fig. 5B). The N-terminal portion of the ZFD forms a regular helix and a 3_10_ helix, which lead up to the first Zn^2+^-coordinating residue C276 in ZnF1. The positions of the two helices are less well defined than the rest of the protein. Thus with only the core rigid portion of the ZFD are superimposed, the RMS deviation for residues 276–336 is decreased to 0.50 ± 0.09 Å for their backbone heavy atoms (Table 1).

**Table 1:** Statistics for the solution structure of METTL3 ZFD

Number of constraints	
NOE	857
Intra (i = j)	556
Short range (|i-j| < 3)	205
Long range (|i-j| ≥ 3)	96
PRE	52
H-bond	32
Dihedral angles	156
φ	65
ψ	60
*χ*1	31
SAXS constraints^#^	2
Zn^2+^-coordination constraints^¶^	32
Structure statistics	
Number of violations (per structure)	
NOE derived constraints (>0.5 Å)	0
PRE derived constraints (>5.0 s^−1^)	0.63
Dihedral angle constraints (>5°)	0
RMS of constraints	
NOE derived constraints (Å)	0.09 ± 0.002
PRE derived constraints (s^−1^)	1.81 ± 0.05
PRE Q-factor	0.09 ± 0.002
Dihedral angle constraints (°)	0.68 ± 0.07
SAXS constraints	0.04 ± 0.002
Average Pairwise r.m.s. deviation (Å)	
Backbone heavy atoms^§^	0.90 ± 0.20 (all)
	0.50 + 0.09 (core)
All heavy atoms	1.22 ± 0.16 (all)
	0.95 ± 0.07 (core)
Ramachandran plot appearance	
Most favored	65.8%
Additionally allowed	26.0%
Generously allowed	6.3%
Disallowed	1.8%

^#^Collected for ZFD constructs spanning residues 259–357 and residues 259–338

^¶^Distance and dihedral angle restraints for the two CCCH-type Zn^2+^-finger (Peters et al. 2010)

^§^The core rigid residues of the ZFD comprise residues 276–336

Though flexible, the C-terminal tail likely folds back onto itself. The folded structure can account for the large PRE values observed for the C-terminal residues 342–352 (Fig. 3C). The partially folded structure can also account for the relatively high *R*_1_ and XNOE values for residues in the tail (Fig. 2B). However, the distance distribution derived from the ZFD SAXS data indicates that the protein has a larger maximum pairwise distance *D*_max_ and thus can be more extended (Fig. 4B). The only explanation to reconcile the discrepancy between NMR PRE and SAXS data is that the ZFD tail alternates between open and closed conformations.

### Mapping the functional interface of ZFD with RNA target

**Figure 6:**
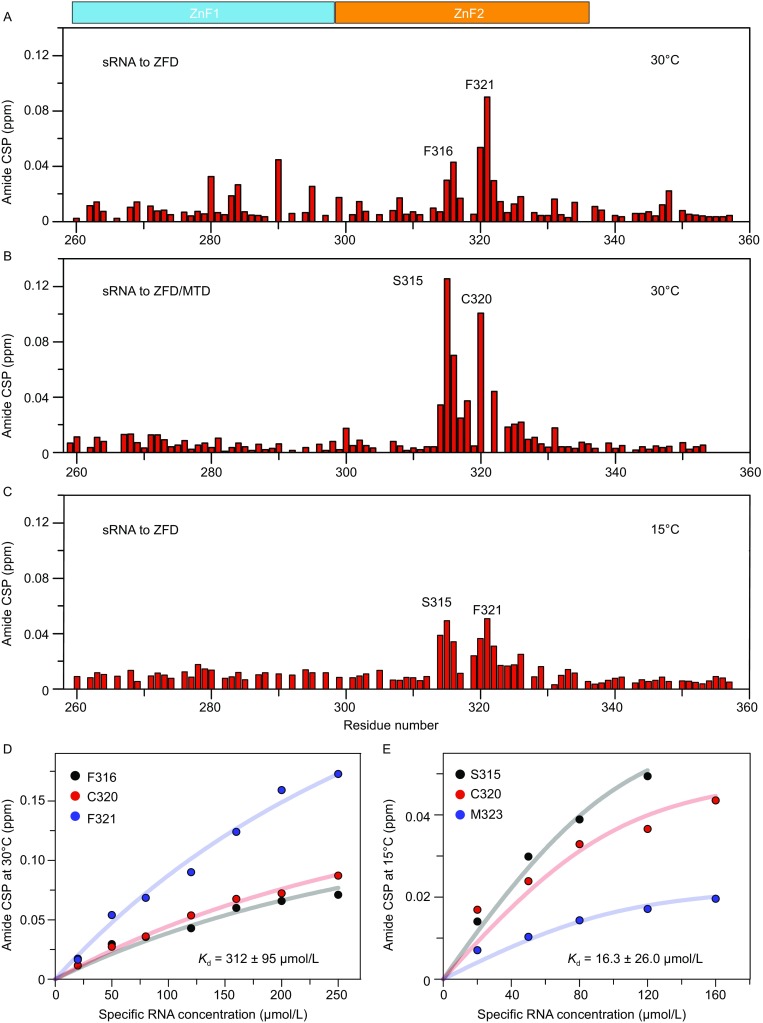
**NMR titrations for METTL ZFD with the specific RNA substrate**. (A and C) Chemical shift perturbations (CSPs) for the backbone amide of 100 µmol/L ^15^N-labeled ZFD mixed with 120 µmol/L 20-nt RNA substrate containing the m^6^A consensus sequence at two different temperatures. Note the NMR experiment could not be performed at 10°C due to severe line broadening of the free protein. No residues disappear upon RNA titration when RNA concetration is lower than 120 μM. Proline, residues with severe signal overlapping or residues without assignment is indicated with a gap in the histogram. (B) The CSPs for ^15^N-labeled ZFD natively ligated to the MTD heterodimer of METTL3-METTL14, upon the titration of specific RNA. The experimental conditions are the same as in panel A except for the protein used. Residues F321 and M323 disappeared in the complex, precluding accurate assessment of their CSPs. (D and E) The *K*_d_ values for the interactions between ZFD and RNA obtained by fitting the CSPs at the two temperatures. Note that molar ratio of RNA relative to ZFD could only reach ~1.6 at the lower temperature

To map the interface between the ZFD with the specific RNA substrate, we performed NMR titrations for the ^15^N-labeled ZFD with an RNA containing the consensus m^6^A methylation sequence 5′-GGACU-3′. At 30°C, the chemical shift perturbations (CSPs) mainly involve the residues in ZnF2, with residues S315, F316, C320, F321 and H322, all located in the hydrophobic patch of ZnF2, experiencing the largest (Figs. S6 and 6A). Significantly, for the selectively isotope-enriched ZFD domain as part of the enzymatically active METTL3-METTL14 heterodimer, titration of the specific RNA elicits similar CSP profile as the isolated ZFD (Fig. 6B). This means that the RNA binding mode is preserved. However, the interaction between ZFD and RNA at 30°C is very weak, with the *K*_d_ value fitted from the CSPs only 312 ± 95 µmol/L (Fig. 6D).

At 15°C, the chemical shift perturbations are smaller (Fig. 6C), though involve similar residues as at 30°C and can also be mapped to the hydrophobic patch in the ZnF2. Note that the NMR signals are much more broadened at 10°C, thus preventing further analysis. In line with the ITC measurement, the protein-RNA interaction is stronger at the lower temperature (Fig. 1C); the *K*_d_ value fitted from NMR CSPs is 16.3 ± 26.0 µmol/L at 15°C (Fig. 6E). The increase in binding affinity at lower temperature further attests that hydrophobicity plays an important role in the interaction between the ZFD and the m^6^A RNA substrate.

To evaluate how the NMR-mapped residues in METTL3 ZFD participate in methyltransferase activity, we introduced alanine mutations to residues 316–323. Mutations to the aromatic residues F316 and F321 have the largest effect on the catalytically active heterodimer comprising METTL3 ZFD-MTD and METTL14 MTD, lowering the enzymatic activity to <10% of the wild-type level (Fig. 5C). L317A and N318A mutations also decrease the activity by more than 50%. Though S315 experiences large CSP upon specific RNA titration, the S315A mutation has a very small effect on the enzymatic activity. On the other hand, alanine mutation to Y331, an aromatic residue in the β-sheet close to the hydrophobic loop, has a large effect on the enzymatic activity of METTL3-METTL14 heterodimer, even though its CSP is not obvious.

We also introduced charge-reversal mutations to basic residues in the ZFD. Introduction of R295D and K296D mutations in the ZnF1 and R300D and R301D mutations in the β-sheet, one at a time, lowers the enzymatic activity to ~10% of the wild-type level (Fig. 5D). In comparison, charge-reversal mutations for R292D in the ZnF1 and K305D in the ZnF2 lower the activity only by ~50% level (Fig. 5D). Residues R295, K296 and R301 form a contiguous surface with the hydrophobic residues, and they likely also contribute to the recognition and interaction with the m^6^A RNA substrate. Indeed, the charge-reversal mutant of the ZFD binds to the specific RNA several times weaker than the wildtype ZFD does (Fig. S3B).

## DISCUSSION

We have presented here the solution structure of the ZFD of METTL3, which comprise two tandem CCCH-type Zn^2+^-fingers. Significantly, we have shown that the ZFD is responsible for specific binding towards m^6^A RNA substrate and for fulfilling the methyltransferase activity of METTL3-METTL14 heterodimer. The ZFD is prevalent among RNA m^6^A methyltransferases in eukaryotes, and our data here thus indicate that the ZFD likely functions as a target recognition domain (TRD), a structural feature that has been previously identified in DNA methyltransferases (Bheemanaik et al. 2006). Befitting to the role of TRD, mutations or deletions of METTL3 ZFD residues diminish or abrogate methyltransferase activity.

The similarity between METTL3 ZFD and the TRDs in DNA methyltransferases is further evidenced by a flexible linker between ZFD/TRD and the methyltransferase domain (Bheemanaik et al. 2006; Song et al. 2011). Interestingly, the solution structure shows that the linker between the ZFD and MTD of METTL3 is partially ordered (Fig. 2B) and adopts a folded-back structure (Fig. 5B). Such a structure should allow the ZFD and MTD to work together when installing an m^6^A mark to an RNA substrate. Furthermore, other protein factors including WTAP, KIAA1429 and RBM15 (Ping et al. 2014; Schwartz et al. 2014; Patil et al. 2016) and post-translational modifications (Zhou et al. 2013) may modulate the structure of METTL3-METTL14 complex and consequently the binding affinity towards certain RNA substrate.

The affinity between METTL3-METTL14 and its RNA substrate turns out extremely weak, with the *K*_d_ value in hundreds of micromolar at physiological temperature. The large equilibrium dissociation constant can be translated to a fast off-rate (Xing et al. 2014), which would enable the enzyme to rapidly scan through potential methylation sites, and account for the high abundance of m^6^A modification in coding and non-coding RNAs (Dominissini et al. 2012; Sun et al. 2016). In this sense, the RNA epigenetic “writer” is fundamentally different from a “reader” for specific RNA target sequence, with the *K*_d_ value typically between nmol/L and low µmol/L (Dominguez et al. 2011; Schlundt et al. 2017).

Based on the solution structure of ZFD, it can be inferred that the ZnF1 interacts with RNA electrostatically, while the ZnF2 interacts with RNA hydrophobically. Indeed, through both NMR chemical shift mapping and mutational analyses, we have identified R295, K296 in the ZnF1, R301 and Y331 in the β-sheet, and F316 and F321 in the ZnF2 as the key residues for RNA interaction. The absence of NMR CSPs for the positively charged residues in ZnF1 upon RNA titration (Fig. 6A) is likely due to similar chemical environment for backbone amide protons when surrounded by water or interacting with RNA, and is also indicative that the interactions between ZnF1 and RNA is mainly electrostatic and likely nonspecific. On the other hand, the inability of NMR CSPs in manifesting the full functional interface of ZFD also tells the fleeting nature of the protein-RNA complex.

## MATERIALS AND METHODS

### Cloning and protein sample preparation

The human *METTL3* gene (GeneBank ID NP_062826.2) was synthesized (GENEWIZ, China). The ZFD, from residue S259 to residue Q357, was amplified and sub-cloned to a pET15d vector (Novagen). A 6× His tag and drICE protease cleavage sequence were appended at the N-terminus of the ZFD. Point mutations in the ZFD of METTL3 were introduced using the QuikChange stratagem, and were verified by DNA sequencing. After transformed into *E*. *coli* strain BL21 (DE3), the cells were grown in either LB medium for preparing unlabeled protein, or M9-minimum medium with ^15^NH_4_Cl and/or ^13^C-glucose (Isotec, Miamisburg, OH) as the sole nitrogen and/or carbon sources for preparing isotope-enriched protein. The culture medium contains 250 µmol/L ZnCl_2_.

The protein expression was induced with 0.5 mmol/L IPTG at OD_600nm_ of 1.0. After induction, the culture temperature was lowered from 37°C to 16°C, and was allowed to grow for another 16 h. The pellet was collected in ice-cold lysis buffer containing 25 mmol/L Tris-HCl pH 8.0 and 150 mmol/L NaCl. The cells were disrupted in a cell homogenizer, and the insoluble fraction was removed with centrifugation at 23,000 ×*g* at 4°C for 1 h.

The supernatant was loaded onto a gravity column of Ni^2+^ affinity resin (Ni-NTA, Qiagen). The resin was washed with a buffer containing 25 mmol/L Tris-HCl pH 8.0, 150 mmol/L NaCl, and 15 mmol/L imidazole. The protein was then eluted with a buffer containing 25 mmol/L Tris-HCl, pH 8.0, and 250 mmol/L imidazole. The protein was further purified with Source-Q anion-exchange chromatography (GE Healthcare). The purification tag from the vector was removed with drICE protease (protein and protease mixed at 100:1 molar ratio) at 4°C for 4 h. With the tag removed, the N-terminus of the ZFD contains three additional residues, AHM, before S259.

The purified ZFD was concentrated to ~10 mg/mL in Amicon Ultra (Millipore), and was further purified with Superdex-200 size-exclusion chromatography (GE Healthcare). The buffer used for the size-exclusion chromatography contained 20 mmol/L pH 6.8 sodium phosphate buffer containing 100 mmol/L NaCl. The peak fractions corresponding to the ZFD were pooled, and used for further study.

The methyltransferase core domains, encompassing residues G360–L580 of METTL3 and Q111–S404 of METTL14, were co-expressed in *E*. *coli* as previously described (Wang et al. 2016). The purification of the MTD heterodimer was performed following the established protocol (Wang et al. 2016). For the measurement of methyltransferase activity, the heterodimers of the full-length METTL3-METTL14 and of ZFD-MTD of METTL3 (residues S259–L580) and MTD of METTL14 were co-expressed and purified.

For the native ligation between the ZFD and MTD domains of METTL3, we modified the last three residues of the ZFD from LTQ to LPQTGGD (evZFD). Fortuitously, the MTD of METTL3 starts with GG (G360 and G361), and therefore no modification of the MTD sequence was needed. The ligation reaction was performed at room temperature in 50 mmol/L pH 8.0 Tris-HCl buffer containing 150 mmol/L KCl and 10 mmol/L CaCl_2_, following the established protocol (Freiburger et al. 2015). The reaction mixture contained 16 µmol/L ^15^N-labeled evZFD, 10 µmol/L unlabeled MTD heterodimer of METTL3-METTL14 (co-expressed and purified), and 20 µmol/L sortase. The sortase with a modified sequence was synthesized by Genewiz, sub-cloned to a pET21d vector (Novagen), and purified as previously described (Antos et al. 2016). Nearly 50% of the evZFD and MTD were found ligated, as assessed on the denaturing gel. The ligation product was purified over Superdex-200 size exclusion column in 20 mmol/L pH 6.8 sodium phosphate buffer 100 mmol/L NaCl, and was assessed for methyltransferase activity.

### RNA sample preparation and m^6^A RNA methylation assay

RNA samples were synthesized using the ABI-3400 Synthesizer with phosphoramidites including 5′-DMT-2′ TBDMS rG (N-iBu), rA (N-Bz), rC (N-Ac), rU (GenePharma, China), following the manufacturer’s standard protocol. The RNA product was cleaved off from CPG matrix, and the protecting group was removed with saturated ammonia at 65°C for 1 h. After lyophilization, TBDMS protecting group was removed with triethylamine trihydrofluoride at 65°C for 2.5 h. The RNA was precipitated with butanol and purified with 15% denaturing PAGE. The bands corresponding to the RNA product were excised from the gel. The RNA product was eluted using Whatman Elutrap (GE Healthcare) in TBE buffer, and was concentrated and buffer-exchanged using Amicon Ultra (Millipore). The specific RNA has the sequence of 5′-AAAAGGACUAAAA-3′, and the nonspecific RNA has the sequence of 5′-AAAAAAAAAAAAA-3′.

For methyltransferase activity measurement, the reaction mixture contained 15 mmol/L HEPES pH 7.3, 50 mmol/L KCl, 50 mmol/L NaCl, 1 mmol/L MgCl_2_, 1 mmol/L DTT, 4% glycerol, 0.04 μCi of [methyl-^3^H] AdoMet (PerkinElmer), 2 nmol/L RNA and 250 ng of METTL3-METTL14 heterodimer in a total volume of 50 μL. The reaction was carried out at 30°C for 1 h. For negative control, the adenine in GGACU consensus sequence was replaced with a guanine. The reaction was quenched with 500 μL of 1:1 (*v*/*v*) Tris-phenol (pH 8.0)/chloroform, and 450 μL of ddH_2_O. The solution was centrifuged at 15,000 ×*g* for 10 min. The supernatant was transferred to a new tube. 600 μL isopropanol (Sinopharm Chemical Reagent), 50 µg of yeast tRNA (Roche), and 120 μmol/L sodium acetate solution (Sigma-Aldrich) were added at −20°C for 1 h to precipitate the methylated RNA product. The precipitated RNA was dissolved in 70 μL of ddH_2_O. The methylation product was confirmed by immune-blot using the commercial m^6^A antibody (Synaptic Systems, catalogue number 202 003, 1:3,000 dilution). The radioactive counts per minute for the methylated RNA were measured using a scintillation counter (1450 MicroBeta Trilux, PerkinElmer). Three independent measurements were performed and the average values (±SD) were reported.

### Isothermal titration calorimetry (ITC) measurements

ITC measurements for the binding affinities between the specific RNA and the protein were performed at 30°C and 10°C using Auto-iTC100 titration calorimetry (MicroCal/Marvin). RNA was dissolved in the reaction buffer containing 20 mmol/L sodium phosphate pH 6.8 (or 20 mmol/L HEPES buffer pH 7.5) and 100 mmol/L NaCl (100 μL in total volume loaded into the syringe), and was titrated into the protein (400 μL in total volume in the cell) that had been extensively dialyzed against the same buffer. The first injection (0.5 μL) was followed by 19 injections of 2 µL each. The heat of dilution for RNA was measured for background subtraction. The titration curves were analyzed with Origin X (MicroCal) with ones-site binding model, with the first injection point excluded. For every combination of protein and RNA, the ITC titrations were repeated three times and the average values (±SD) were reported. ITC titrations were also performed the charge-reversal mutant of the ZFD in pH 6.8 sodium phosphate buffer at 10°C.

### NMR data collection and analyses

The protein for the ZFD (residues 259–357 of METTL3) was isotopically labeled with ^15^N or ^13^C/^15^N, and was prepared in 20 mmol/L pH 6.8 sodium phosphate buffer containing 100 mmol/L NaCl (with 10% *v*/*v* D_2_O added). The NMR data were acquired at 30°C on Bruker 600 MHz or 850 MHz spectrometers equipped with cryogenic probes. Standard triple resonance experiments including HNCO, HNCACB and CBCACONH, and HCCONH and CCONH experiments were used to assign backbone and side resonance, respectively. NMR data were processed with NMRPipe (Delaglio et al. 1995), and the triple resonance spectra were analyzed with CCPN Analysis Version 2.4.2. HCBCGCDHD experiment (Yamazaki et al. 1993) was recorded for the assignment of the aromatic side chains. 2D long-range ^15^N-HSQC experiments were performed to identify tautomeric states of histidine residues (Pelton et al. 1993). The center of the spectrum was set at 200 ppm, the spectral width at 120 ppm, and the INEPT transfer time at 22 ms.

To assess the *ps*-*ns* dynamics of the ZFD, we performed relaxation analyses for backbone amide nitrogen atoms at Bruker Avance III 600 MHz at 30°C, following the established method (Kay et al. 1989). The XNOE experiment was recorded in an interleaved mode—a total of 8-s recovery delay was applied for the reference experiment, while a 5-s recovery delay followed by 3-s proton saturation was applied for the NOE experiment. The ^15^N T_1_ and T_2_ experiments were also recorded in an interleaved mode. For the T_2_ experiments, the delays were set at 16.96 ms, 50.88 ms, 84.80 ms and 135.68 ms, and for the T_1_ experiments, the delays were set at 5 ms, 100 ms, 300 ms and 500 ms.

A series of ^1^H-^15^N HSQC of ^15^N isotopically labeled ZFD was titrated with increasing concentrations of the specific RNA at 30°C and 15°C. The assignment at 15°C was accomplished by monitoring gradual peak shifts in the HSQC spectra. The spectrum at 10°C was too broad to be unambiguously assigned, and therefore the CSPs at 15°C were used instead. The concentration of ZFD was 100 μmol/L, and the RNA substrate was added to a molar ratio of up to 2.5 folds of the ZFD. Due to severe line broadening at 15°C, the RNA molar ratio could only reach 1.6 for precise chemical shift measurements. The chemical shift perturbation was calculated using the equation [0.5 × (ΔδH^2^ + 0.2 × ΔδN^2^)]^0.5^, in which ΔδH and ΔδN are the CSP in proton and nitrogen dimensions in ppm unit, respectively.

### SAXS data collection and analyses

The SAXS data were collected for the ZFD with three additional residues AHM appended at the N-terminus. A truncated version of ZFD was also prepared, which spans residues 259–338, with the last 20 flexible residues removed. In this way, the scattering contribution from the C-terminal tail did not have to be considered. The protein was dialyzed extensively against the same buffer used for NMR, and the background diffraction from the buffer was collected and subtracted. The inverse Fourier transformation of the scattering profile to paired distance distribution was performed with PRIMUSQT. The fitting of the ZFD structure to the scattering profile was performed using CRYSOL. PRIMUSQT and CRYSOL are part of the ATSAS 2.8 suite (Franke et al. 2017). The calculation of paired distance distribution for the final structures was performed using AMBER 14 (Case et al. 2014).

### Paramagnetic labeling of the ZFD

The ZFD protein carrying D334C/C336S double mutation was purified, and buffer-exchanged into pH 7.2 HEPES buffer containing 100 mmol/L NaCl and 2 mmol/L DTT. Maleimide-EDTA (cat no. P138480, purchased from TRC, Canada) was first mixed with MnCl_2_ stock solution. The protein, upon desalting to remove the DTT, was allowed to react with maleimide-EDTA at room temperature for 2 h. The molar ratio for Mn^2+^, maleimide-EDTA and the protein was 6:3:2. Carrying one more negative charge than untagged protein, the paramagnetically tagged ZFD protein was purified with Source-Q chromatography, and was confirmed by ESI-MS (Bruker Daltonics, Billerica MA). The intra-molecular transverse relaxation rates Γ_2_ of the tagged protein was measured at 30°C with the standard ^1^H-^15^N HSQC based pulse sequence (Iwahara et al. 2007), with the wild type protein as diamagnetic reference.

### Structure calculation

The ^15^N- or ^13^C-edited 3D NOESY-HSQC spectra were acquired with 120 ms NOE mixing time. The NOE cross-peaks in the 3D NOESY spectra were auto-assigned using the software ARIA (Rieping et al. 2007). In addition, 2D NOESY was collected for an unlabeled ZFD prepared in 100% D_2_O buffer, and the NOE cross-peaks were manually assigned mainly involving aromatic side chains. The structural calculation was performed in Xplor-NIH (Schwieters et al. 2006) using the standard protocol, by refining against NOE distance restraints (from both 3D and 2D NOESY datasets), backbone dihedral angle restraints (predicted using the software TALOS+ (Shen et al. 2009) from backbone chemical shift values), the PRE restraints, and small-angle X-ray scattering (Schwieters and Clore 2014) restraints.

Based on the long-range ^15^N-HSQC data, knowledge-based restraints were also applied to C276, C284, C294 and H298 for the first zinc finger and to C314, C320, C326 and H330 for the second zinc finger, thus to enforce the geometry for Zn^2+^ coordination (Peters et al. 2010; Sun et al. 2016), with the Nε2 atoms of H298 and H330 are directly bonded to Zn^2+^. The TALOS+ predicted dihedral angle restraints were not applied to Zn^2+^-coordinating residues and their adjacent residues, which we found would otherwise cause constant NOE violations. The SAXS restraints, collected for both ZFD (for residues 259–357) and the truncated version of ZFD (for residues 259–338), were applied.

The maleimide-EDTA-Mn^2+^ paramagnetic probe was patched to D334C/C336S mutant of ZFD. As the thioether bond can be formed in two alternative configurations (Liu et al. 2012), we represented each covalent configuration with three conformers for a total of the six conformers for the probe. Different conformers in the ensemble are allowed to overlap, thus to recapture all possible conformational space. The Mn^2+^ ion has an electron relaxation time τ_e_ of 9.6 ns. Estimated from ^15^N R_2_ and R_1_ relaxation rates, the protein has an apparent rotational correlation time τ_c_ of 12 ns. Thus the PRE correlation time is estimated at 5.3 ns. The *N* = 3 × 2 ensemble of the paramagnetic probe is optimized, while at the same time when the protein structure is refined. Importantly, the incorporation of the PRE restraints does not incur additional violations for NOE or backbone dihedral angle restraints. The C-terminal tail of the ZFD (residues 339–354) was also applied with the PRE restraints. As these residues are dynamic and have a smaller PRE correlation time, a square-welled energy function was used with a large upper bound for the PRE restraints and model-free analysis of the Solomon-Bloembergen equation was used to account for additional scaling down of the PRE value.

A total of 256 structures were calculated. The structures with fewest violations and lowest energy were further assessed. To illustrate electrostatic potential surface of the ZFD, the PDB file was assigned with partial charges using pdb2pqr with the parameters from AMBER force field. The structures were assessed with PROCHECK (Laskowski et al. 1996), and structure figures were rendered in PyMOL (The PyMOL Molecular Graphics System) (Schrödinger Inc.).

## DATA AVAILABILITY

Coordinates and NMR restraints have been deposited at the PDB with the accession code 5YZ9 and at the BMRB with the accession code 36143.

## Electronic supplementary material

Below is the link to the electronic supplementary material.

Supplementary material 1 (PDF 941 kb)
