# Supplementary material for: Solution structure of the RNA recognition domain of METTL3-METTL14 N6-methyladenosine methyltransferase
Source: Protein Cell. 2018 Mar 14;10(4):272–84. doi: 10.1007/s13238-018-0518-7 (PMC6418081; doi:10.1007/s13238-018-0518-7)
Supplement: Supplementary file 1 — Supplementary material 1 (PDF 941 kb) [file 13238_2018_518_MOESM1_ESM.pdf]

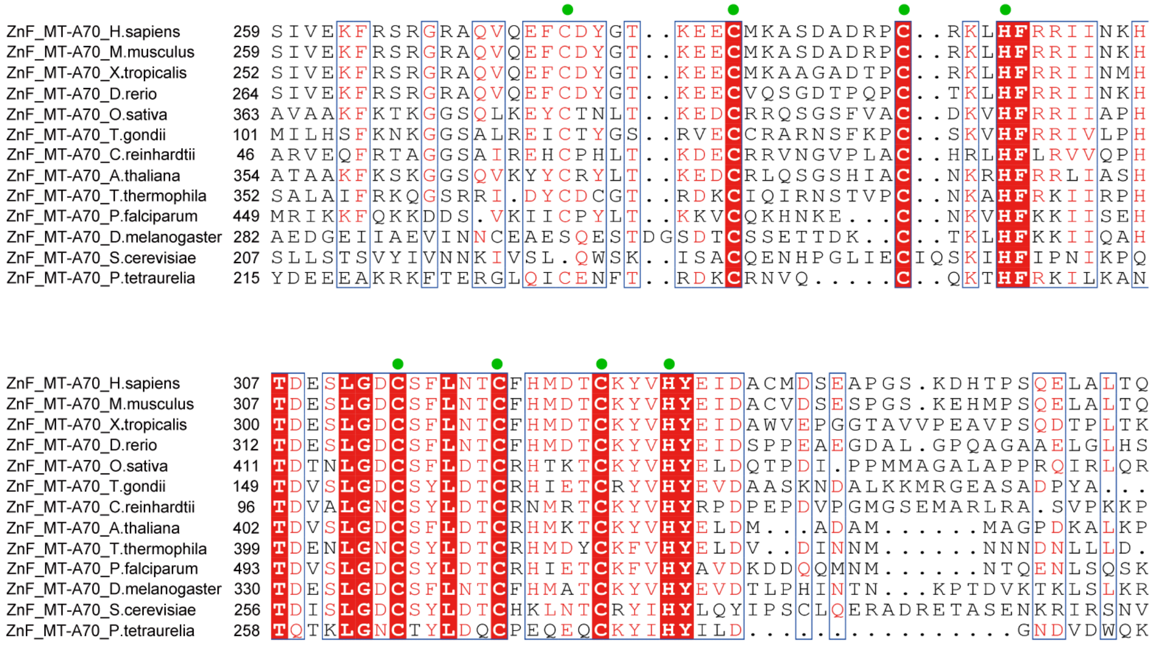

Supplementary Figure 1. Sequence alignment of the Zinc-finger domain of METTL3 from different species. Red highlight indicates residues that are strictly conserved, and red font indicates residues that are mostly conserved (>70%). The second Zn^{2+}-finger appears more conserved than the first Zn^{2+}-finger. Green dots denote residues that are involved in Zn^{2+} coordination. The N^6-adenosine-methyltransferase 70-kDa subunit is denoted here as MT-A70. Species abbreviation and GeneBank accession ID: H.sapiens, *homo sapiens*, NP_062826.2; M.musculus, *Mus musculus*, NP_062695.2; X.tropicalis, *Xenopus tropicalis*, NP_001016419.1; D.rerio, *Danio rerio*, NP_997945.1; O.sativa, *Oryza sativa*, XP_015625439.1; T.gondii, *Toxoplasma gondii*, KFG50663.1; C.reinhardtii, *Chlamydomonas reinhardtii*, XP_001691478.1; A.thaliana, *Arabidopsis thaliana*, NP_192814.1; T.thermophila, *Tetrahymena thermophila*, EAS00013.2; P.falciparum, *Plasmodium falciparum*, XP_001349190.1; D.melanogaster, *Drosophila melanogaster*, NP_651204.1; S.cerevisiae, *Saccharomyces cerevisiae*, CAA96904.1; P.tetraurelia, *Paramecium tetraurelia*, XP_001450303.1.

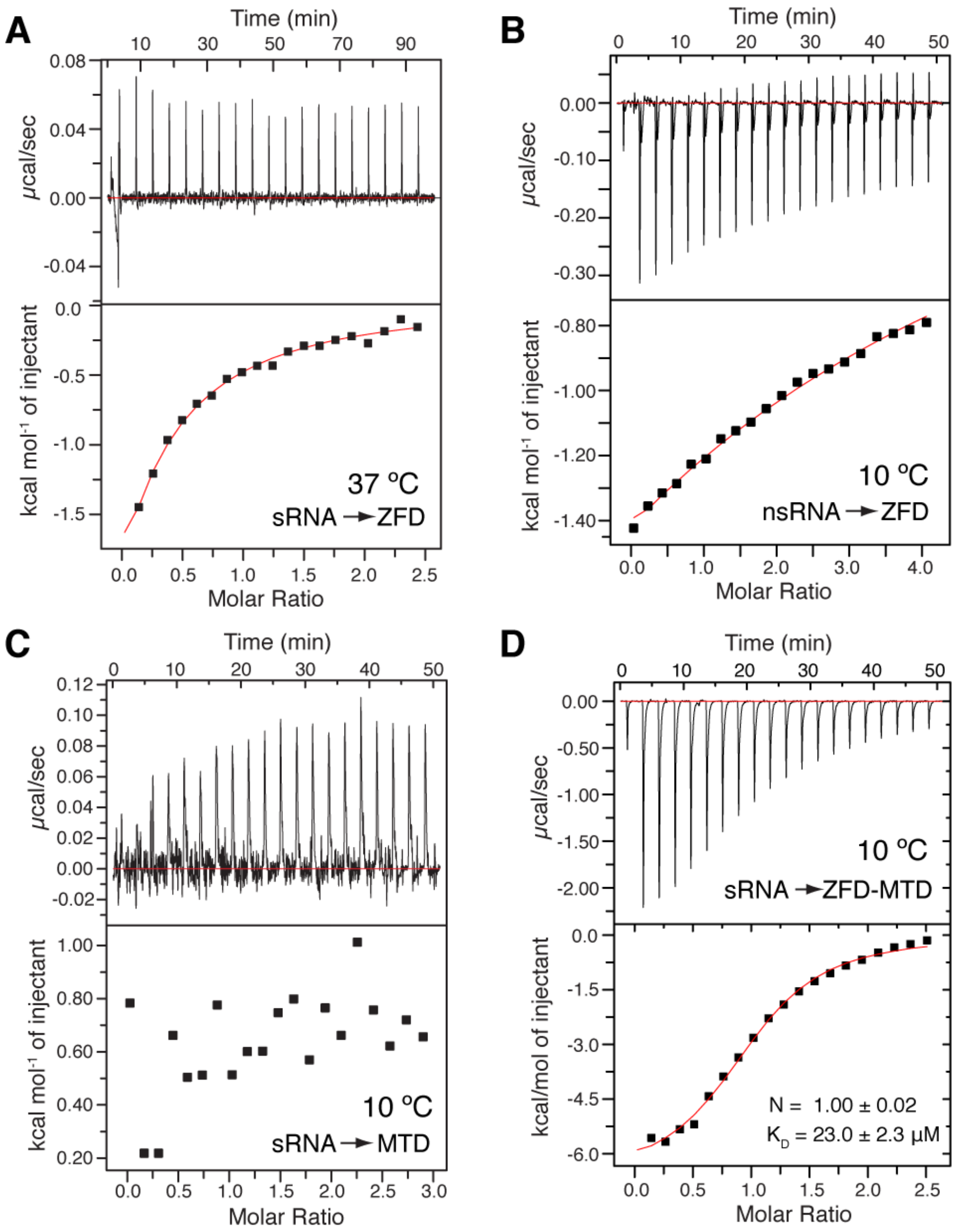

Supplementary Figure 2. Isothermal calorimetry (ITC) measurements for RNA-protein interaction. The titrations were conducted at either 37 ºC or 10 ºC otherwise under the same conditions as in Figure 1C. (**A**) A 1.5 mM specific RNA containing 5′-GGACU-3′ sequence was titrated to 70 µM ZFD at 37 ºC. Though the ITC curve can be arbitrarily fitted ($K_D \approx$ 120 µM), the stoichiometry is way off (0.005). (**B**) At 10 ºC, a 20-nt nonspecific RNA containing only adenosines (4 mM) was titrated to the ZFD of METTL3 (200 µM). Though the ITC data can be arbitrarily fitted ($K_D \approx$ 123 µM), the stoichiometry is off (N = 2.05). (**C**) A 1 mM specific RNA substrate was titrated to the heterodimer of METTL3-METTL14 (70 µM). In this case, the binding isotherm was too weak to be assessed. (**D**) A specific RNA (2 mM) was titrated to the enzymatically active heterodimer comprising the METTL3 ZFD-MTD and METTL14 MTD (200 µM). The isotherm can be fitted using a one-site binding model to a K_D value of ~23 µM. All ITC experiments were repeated at least three times.

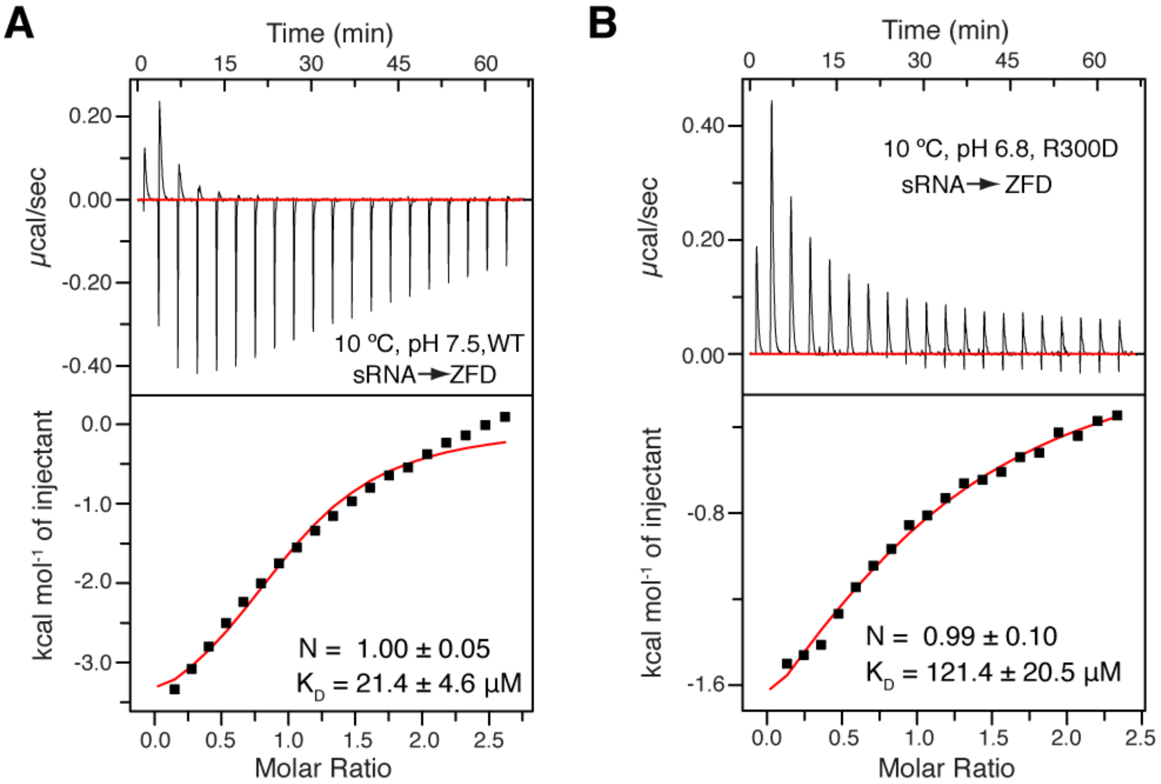

Supplementary Figure 3. ITC titrations for measuring the affinities between METTL3 ZFD and specific RNA. The experiments were done at 10 ºC, with 1.38 mM specific RNA titrated into 100 µM ZFD protein. (**A**) The titration was performed at 20 mM pH 7.5 HEPES buffer containing 100 mM NaCl. The ITC profile and fitted K_D value is almost identical to those performed at pH 6.8 (c.f. Fig. 1C). (**B**) The titration was performed at 20 mM pH 6.8 phosphate buffer containing 100 mM NaCl for R300D charge reversal mutant. The K_D value is almost 6 times weaker for the mutant.

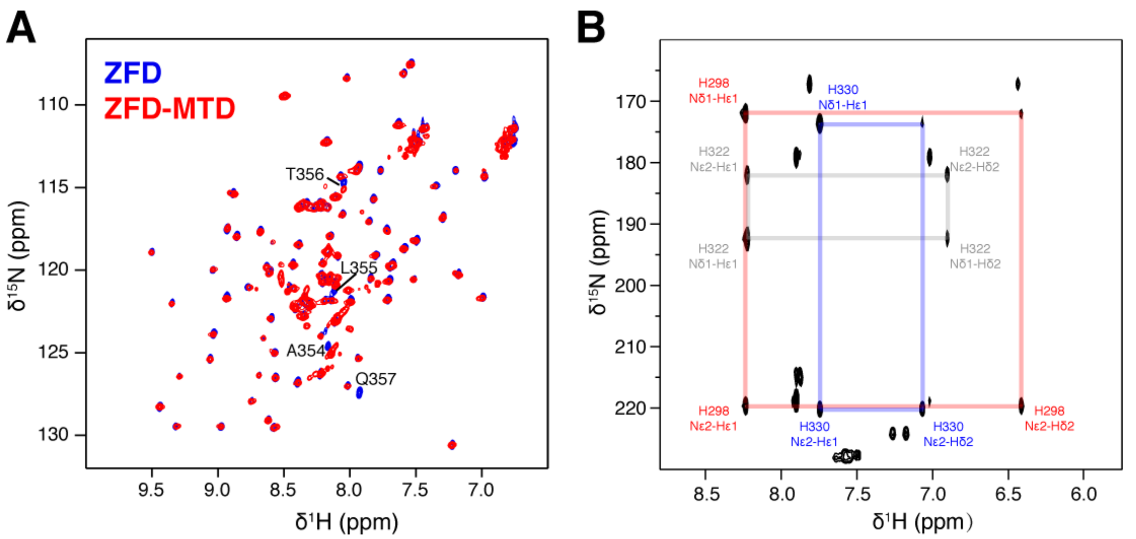

Supplementary Figure 4. ^{1}H-^{15}N HSQC spectra of the ZFD of METTL3. (**A**) Overlay of the ^{1}H-^{15}N HSQC spectra for the isolated ZFD and segmentally ^{15}N-labeled ZFD as part of the METTL3-METTL14 catalytically active heterodimer. Except for a few residues near the ligation site (labeled), the two spectra can be nicely overlaid. (**B**) Long-range ^{1}H-^{15}N HSQC spectrum for assessment of the tautomeric states of the histidines. A carbon-attached proton in the histidine imidazole ring (Hε1 or Hδ2) can be correlated to imidazole nitrogen (Nε2 or Nδ1) through a two-bond scalar coupling. If the nitrogen is protonated, the cross-peak can be effectively dephased over a 22-ms INEPT transfer period. Illustrated with red and blue rectangles respectively, H298 and H330 exist in δ1-H neutral tautomeric states, meaning that Hδ1 is present while Hε2 is absent, available for Zn^{2+}-coordination. In comparison, H322 exists in the charged form (gray rectangle).

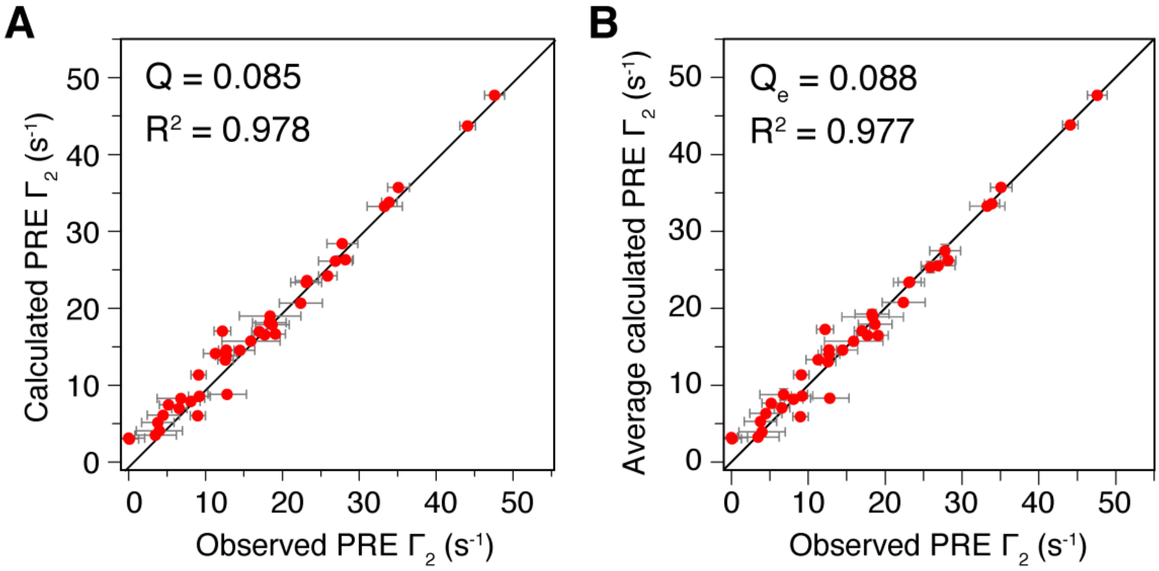

Supplementary Figure 5. The correlation between observed and back-calculated PRE Γ_2 values for the backbone amide protons of the ZFD. (**A**) The PRE values are calculated for the conformer with the lowest PRE energy in the bundle. (**B**) The PRE values are calculated for all 25 conformers in the bundle. The error bars indicate 1 S.D. in the measurement uncertainty for the observed values, and indicate 1 S.D. when averaging the calculated values from all conformers in the structure bundle. Only the PRE data for the rigid residues (before S338) are used to plot the correlation. The correlation coefficient and PRE Q-factors (Qe for the bundle) are reported.

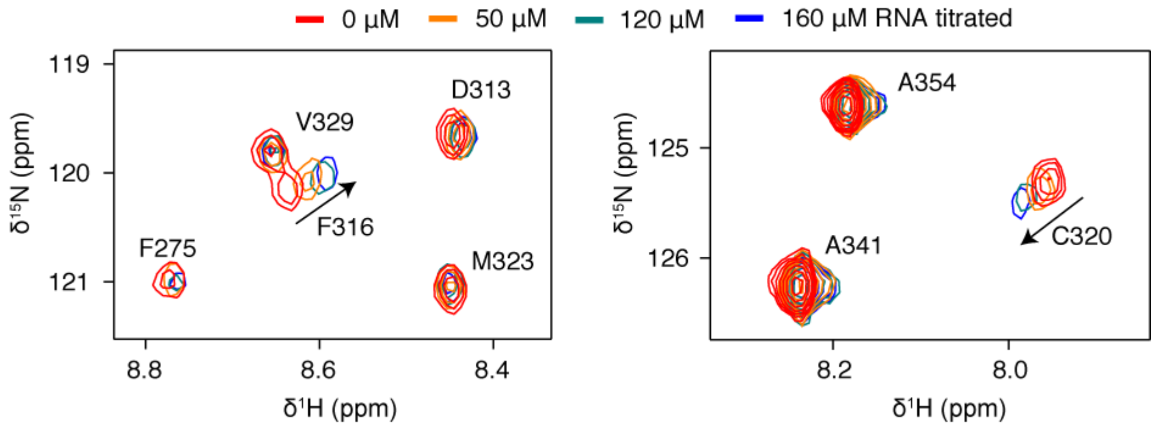

Supplementary Figure 6. Overlay of the 2D NMR HSQC spectra for ^{15}N-labeled METTL3 ZFD titrated with increasing concentrations of the specific RNA. The two sleeted regions show the progressive shift of the peaks for F316 and C320, both located at the interface, upon RNA binding. In comparison, peaks for residues away from the interface display no shifts.
